# A new role for *muscle segment homeobox* genes in mammalian embryonic diapause

**DOI:** 10.1098/rsob.130035

**Published:** 2013-04

**Authors:** Jeeyeon Cha, Xiaofei Sun, Amanda Bartos, Jane Fenelon, Pavine Lefèvre, Takiko Daikoku, Geoff Shaw, Robert Maxson, Bruce D. Murphy, Marilyn B. Renfree, Sudhansu K. Dey

**Affiliations:** 1 Division of Reproductive Sciences, Cincinnati Children's Research Foundation, Cincinnati, OH 45229, USA; 2 Center for Animal Reproduction, Faculty of Veterinary Medicine, University of Montréal, Saint Hyacinthe, Québec, CanadaJ2S 7C6; 3 Department of Zoology, University of Melbourne, Melbourne, Victoria 3010, Australia; 4 Department of Biochemistry and Molecular Biology, Keck School of Medicine, Norris Comprehensive Cancer Center and Hospital, University of Southern California, Los Angeles, CA 90033, USA

**Keywords:** MSX genes, delayed implantation, embryonic diapause, mouse, mink, tammar wallaby

## Abstract

Mammalian embryonic diapause is a phenomenon defined by the temporary arrest in blastocyst growth and metabolic activity within the uterus which synchronously becomes quiescent to blastocyst activation and implantation. This reproductive strategy temporally uncouples conception from parturition until environmental or maternal conditions are favourable for the survival of the mother and newborn. The underlying molecular mechanism by which the uterus and embryo temporarily achieve quiescence, maintain blastocyst survival and then resume blastocyst activation with subsequent implantation remains unknown. Here, we show that uterine expression of *Msx1* or *Msx2*, members of an ancient, highly conserved homeobox gene family, persists in three unrelated mammalian species during diapause, followed by rapid downregulation with blastocyst activation and implantation. Mice with uterine inactivation of *Msx1* and *Msx2* fail to achieve diapause and reactivation. Remarkably, the North American mink and Australian tammar wallaby share similar expression patterns of *MSX1* or *MSX2* as in mice*—*it persists during diapause and is rapidly downregulated upon blastocyst activation and implantation. Evidence from mouse studies suggests that the effects of *Msx* genes in diapause are mediated through Wnt5a, a known transcriptional target of uterine *Msx*. These studies provide strong evidence that the *Msx* gene family constitutes a common conserved molecular mediator in the uterus during embryonic diapause to improve female reproductive fitness.

## 2. Introduction

Embryonic diapause is a transient state in which embryos at the blastocyst stage are arrested in growth and metabolic activity in synchrony with uterine quiescence. This provisional delay of implantation allows pregnancy to withstand harsh environmental conditions, or adapt to a changing photoperiod or maternally derived stimuli such as lactation, thereby allowing pregnancy to resume during conditions favourable to rear young [1–4]. This reproductive strategy is widespread, occurring in nearly 100 mammalian species across seven orders [1,2].

Although features of diapause vary across species with respect to endocrine status [1,2,4], the presumed purpose is to prolong the gestational period, allowing young to be born at optimal times of the year with respect to a species’ nutritional and environmental needs and apportioning the mother's metabolic resources to nurse sequential litters. The pervasive existence of mammalian embryonic diapause implies that the pressure to safeguard and preserve procreation under diverse conditions through sexual reproduction is one of the fundamental forces in mammalian evolution. However, the underlying molecular mechanism by which the uterus and embryo temporarily achieve growth restriction and then resume blastocyst activation for implantation under favourable conditions remains largely unknown.

Delayed implantation can occur naturally during lactation after postpartum mating (facultative delay). The embryo develops to the blastocyst stage and remains dormant within the quiescent uterus, while the newly born pups are suckling, allowing sufficient time to nurse sequential litters. In mice, this stems from heightened levels of pituitary prolactin from the suckling stimulus that attenuates ovarian oestrogen secretion [5,6]; removing this stimulus can trigger blastocyst reactivation (implantation competency) and implantation. This strategy is also present in several diapausing mammals, including the Australian tammar wallaby [3]. In this case, prolactin is luteostatic, rendering the corpus luteum quiescent and thus inhibiting progesterone (P_4_) secretion required for uterine secretory activity and reactivation. Removal of the suckling pouch young after the summer solstice induces reactivation. Tammars also display a seasonal diapause (obligatory) controlled by photoperiod and melatonin secretion after the winter solstice [3]. Mink, ferret and skunk, among others, also have seasonal diapause, implicating day length and melatonin levels in regulating diapause via prolactin [2]. Irrespective of the type of delay, embryonic diapause is under maternal regulation in all species studied to date. Although the endocrine milieu behind various natural inducers has been characterized in several diapausing species [1,2,4], the molecular mechanism by which it promotes synchronized uterine quiescence and blastocyst dormancy, while maintaining implantation competency is poorly understood.

Our recent studies in mice show that *muscle segment homeobox* (*Msx*) genes play critical roles in preparing the uterus to the receptive state for blastocyst implantation during normal pregnancy. The expression is transient, appearing on the morning of day 4 (the day of uterine receptivity) and then being dramatically downregulated approaching implantation and thereafter [7]. By contrast, we show here that *Msx1* is highly expressed in the mouse uterine epithelium during embryonic diapause induced by various approaches and becomes undetectable with the initiation of implantation. More importantly, we also found that mice with conditional uterine inactivation of *Msx* (*Msx1* and *Msx2*) have drastically reduced blastocyst recovery and survival. These results suggested that *Msx* genes are critical for conferring true dormancy and implantation competence. These intriguing results led us to study the role of *Msx* genes in embryonic diapause and delayed implantation in representative species of three mammalian orders: mice (Eutheria: Rodentia), American mink (Eutheria: Carnivora) and Australian tammar wallabies (Marsupialia: Diprotodontia). We observed that *Msx* genes share this conserved role in unrelated diapausing mammals. *MSX1* expression persists in the luminal and glandular epithelia of American mink (*Neovison vison*) under obligate delay regulated by photoperiod. The expression is downregulated with the resumption of blastocyst activation and implantation. Similarly, another family member, *MSX2* [8], remains highly expressed in the uterus of the Australian tammar wallaby (*Macropus eugenii*) undergoing lactational diapause, but again is downregulated upon exit from diapause and is absent after blastocyst attachment. As expression of *Msx* genes persists during the delay in these different species, and because mice with uterine deletion of *Msx* genes fail to undergo true delay, we propose that *Msx* genes constitute a gene family whose function is conserved not only for normal implantation, but is also for the maternal regulation of mammalian embryonic diapause.

## 3. Material and methods

### 3.1. Animals

#### 3.1.1. Mice

Mice with uterine deletion of *Msx1* and *Msx2* (*Msx1/Msx2^loxP/loxP^ Pgr^Cre/+^* = *Msx1/Msx2*^d/d^) and control littermates (*Msx1/Msx2^loxP/loxP^ Pgr^+/+^* = *Msx1/Msx2*^f/f^) were generated as previously described [7]. *Msx1*^LacZ^, *Lif^−/−^* and *Pgr-Cre* mice were initially provided by Robert Benoit, Phillippe Brulet, and John Lydon and Francesco DeMayo, respectively [7,9,10]. All mice used in this investigation were housed in barrier facilities in the Cincinnati Children's Hospital Medical Center's Animal Care Facility according to National Institutes of Health and institutional guidelines for the use of laboratory animals. All protocols for this study were reviewed and approved by the Cincinnati Children's Research Foundation's Institutional Animal Care and Use Committee. Mice were provided with autoclaved rodent LabDiet 5010 (Purina) and UV-light-sterilized RO/DI (reverse osmosis/deionized) constant circulation water ad libitum.

#### 3.1.2. Mink

All procedures involving live animals were approved by the University of Montreal Animal Care Committee, which is accredited by the Canadian Council on Animal Care. Investigations were carried out during two consecutive annual breeding seasons in 2011 and 2012 using ranch mink of the Pastel variety acquired from a local farm (A. Richard, Saint Damase, Québec, Canada). During the mink breeding season, which begins in early March, each female was bred to two fertile males according to usual farm mating practices. Uteri were collected in the delayed state, 7–9 days after the final mating, and diapause confirmed by the occurrence of blastocysts in the reproductive tract [11]. In a subset of animals, embryo reactivation was synchronized by daily injection of 1 mg kg^−1^ per day ovine prolactin (Sigma-Aldrich, Oakville, Ontario, Canada) [12] beginning on 21 March and continuing for the following 10 days. The first day of prolactin injection was designated day 0 of embryo reactivation. Uterine horns were collected at days 0 (diapause), 9 and 14 after prolactin-induced reactivation. Implantation occurred approximately 12 days after initiation of prolactin treatment, and uteri with implantation sites were collected at day 14. Tissues were snap-frozen and stored at −80°C until they were shipped to Cincinnati Children's Research Foundation on dry ice for *in situ* hybridization and immunofluorescence analyses.

#### 3.1.3. Tammar wallaby

Tammar wallabies (*M. eugenii*) of Kangaroo Island origin were kept in open grassy yards in a breeding colony maintained by the University of Melbourne. Their diet was supplemented with fresh fruit, vegetables and lucerne cubes, and water was supplied ad libitum. Care and treatment of animals conformed to the National Health and Medical Research Council Australian 2004 guidelines. All animal handling and experimentation were approved by the University of Melbourne Animal Experimentation Ethics Committees. Tammar uterine samples were obtained as previously described [13]. Before and during embryonic diapause, the stage of pregnancy was based on the postpartum age of the pouch young (day 0–day 350 postpartum). Females were checked regularly for births, and if the date of birth was not known, then the ages of pouch young were extrapolated from growth curves based on head length measurements [14]. In this study, adult females with a pouch young older than day 8 postpartum were presumed to be carrying a diapausing blastocyst [3]. Before reactivation from diapause can occur, the pouch young-induced luteal inhibition must be removed for three consecutive days [15,16]. Therefore, after diapause, the reactivation stages of pregnancy were determined relative to the day of the removal of the pouch young (days after RPY). All tissues were collected under RNase-free conditions. Snap-frozen tissues were stored at −80°C, and shipped via cryoshipper in liquid N_2_ to Cincinnati Children's Research Foundation for *in situ* hybridization, immunostaining and qPCR.

#### 3.1.4. Analysis of delayed implantation and activation in mice

Littermate *Msx1/Msx2*^f/f^ and *Msx1/Msx2*^d/d^ females were mated with wild-type (WT) fertile males to induce pregnancy. To induce delayed implantation, mice were ovariectomized on day 4 of pregnancy and administered progesterone (P_4_, 2 mg per dose, sc) in sesame oil until day 8 of pregnancy (4 days of dormancy) or day 10 (6 days of dormancy). Anti-oestrogen (ICI 182,780, Zeneca Pharmaceuticals, 50 µg 100 μl^−1^ dose, sc) was administered on day 3 at 1800 h and day 4 between 0800 and 0900 h to induce delay. Uteri were flushed with physiological saline to recover blastocysts. For activation, mice were injected either with recombinant leukaemia inhibitory factor (LIF) (50 μg per mouse, ip) on day 6 at 1000 and 1600 h or with 25 ng oestradiol-17β (E_2_). Implantation sites were examined 24 or 48 h later. Implantation sites were visualized by intravenous injection of a Chicago blue dye solution, and implantation sites, demarcated by distinct blue bands, were collected. To study lactational delay, pregnant females were housed with males on day 17 of pregnancy onwards and checked for vaginal plugs on days 19–20 [5,6,17]. Upon delivery (P1), pups were either removed or kept with the mother to suckle for 6 days, and uteri were collected 6 days postpartum (P6) for *in situ* hybridization. Delay in implantation during lactation depends on the intensity of suckling stimuli; there is often failure of delay if only a few pups are allowed to suckle [18]. Therefore, only dams with six to eight pups per litter were used in this study.

### 3.2. Hormone treatment

Non-pregnant WT or *Msx1*^LacZ^ female mice were ovariectomized and allowed to recover for 10 days. Females were administered subcutaneously 100 µl of sesame oil, 2 mg P_4_, 25 ng E_2_, or P_4_ + E_2_, and uteri were collected 24 h later and snap-frozen for *in situ* hybridization. Tissues from each treatment were processed onto the same slide. Littermate *Msx1/Msx2*^f/f^ and *Msx1/Msx2*^d/d^ females were administered 2 mg P_4_ on day 3 and day 4 of pregnancy. The morning of finding the vaginal plug was considered day 1 of pregnancy. Implantation sites were visualized by intravenous injection of a Chicago blue dye solution, and implantation sites were counted [19,20]. If no sites were found, then uteri were flushed with physiological saline to recover blastocysts to confirm pregnancy.

### 3.3. Immunostaining

Immunohistochemistry on mouse tissues was performed in formalin-fixed, paraffin-embedded sections using specific antibodies. Primary antibodies for cyclooxygenase-2 (Cox2) (laboratory generated, 1 : 200), cytokeratin 8 (Developmental Studies Hybridoma Bank, 1 : 200), Msx1/2 (recognizes Msx1 and Msx2, Developmental Studies Hybridoma Bank, 1 : 50), Msx1 (Santa Cruz, 1 : 200), phospho-histone H3 (Santa Cruz, 1 : 200) and Ki67 (Neomarkers, 1 : 300) were used. In tammar wallaby studies, tissues were fixed in 4 per cent paraformaldehyde and paraffin-embedded. To compare intensity of immunostaining between tissues, tissue sections from both genotypes on specific day of pregnancy or differential stages of diapause and reactivation were processed on the same slide. A Histostain-Plus (diaminobenzidine) kit (Invitrogen) was used to visualize specific antigens. Immunofluorescence for Msx1 on mink tissues was performed using Msx1 antibody (Santa Cruz), and secondary antibodies Cy2 conjugated donkey anti-rabbit (Jackson Immuno Research) on frozen sections. Nuclear staining was performed using DAPI. Immunofluorescence was visualized with confocal microscopy (Nikon Eclipse TE2000).

### 3.4. *In situ* hybridization

*In situ* hybridization was performed as previously described [21]. Uterine samples from mouse, mink and tammar wallaby were collected and snap-frozen. Uterine sections (12 µm thickness) were mounted onto poly-l-lysine-coated slides, fixed in cold 4 per cent paraformaldehyde, acetylated and hybridized at 45°C for 4 h in formamide hybridization buffer containing species-specific ^35^S-labelled cRNA probes. RNase A-resistant hybrids were detected by autoradiography with Kodak NTB-2 liquid emulsion. Parallel sections were hybridized with the corresponding sense probes. To compare mRNA patterns and intensities, sections of uteri from control and experimental groups or from different days of diapause and reactivation were placed onto the same slide and processed for hybridization. Mouse-specific cRNA probes for *Msx1, Hoxa10, Ihh, Ptgs2, Bmp2* and *Wnt5a* were used for hybridization. For mink and tammar wallaby experiments, dog-specific cRNA probes for *MSX1* and *WNT5A* and tammar wallaby-specific cRNA probe for *MSX2*, respectively, were generated from published genomic sequences.

### 3.5. RT-PCR and qPCR

In mink samples, the housekeeping gene *GAPDH* (encoding glyceraldehyde-3-phosphate dehydrogenase) was used as an internal control. qPCR was performed using primers: 5′-AAGTTCCGCCAGAAGCAGTA-3′ and 5′-AGCCATCTTCAGCTTTTCCA-3′ for *MSX1* (annealing temperature 68°C, product size 220 bp); 5′-AAAGGCAGTGACCTGTT-3′ and 5′-GGTGGGGCTCATATGTCT-3′ for *MSX2* (expected product size 381 bp); and 5′-TCCCCACCCCCAATGTG-3′ and 5′-CCCTCTGATGCCTGCTTCA-3′ for *GAPDH*. In tammar wallaby samples, the housekeeping gene *ACTB* (encodes β-actin) was used as an internal control, and RT-PCR was performed at 35 cycles. RT-PCR and qPCR were performed using primers: 5′-TTG CTG ACA GGA TGC AGA AG-3′ and 5′-AAA GCC ATG CCA ATC TCA TC-3′ for tammar wallaby-specific *ACTB* (annealing temperature 60°C; product size 247 bp); 5′-CCGAAAGTCCTGAGAAGCAG-3′ and 5′-GAGGCTCAGAGAGCTGGAGA-3′ for wallaby-specific *MSX1* (expected product size 233 bp); and 5′-TCGCAGGGTCAAAGTGTCTAG-3′ and 5′-GTTGTGGGACTCAAGTGTCTT-3′ for wallaby-specific *MSX2* (annealing temperature 63°C; product size 311 bp). Primers were generated from the published tammar wallaby sequence and dog sequence.

### 3.6. Statistics

Statistical analyses were performed, using two-tailed Student's *t-*test. A value of *p* < 0.05 was considered statistically significant.

## 4. Results

### 4.1. *Msx1* expression persists in the mouse uterine epithelium during delayed implantation induced by multiple approaches

**Figure 1.**
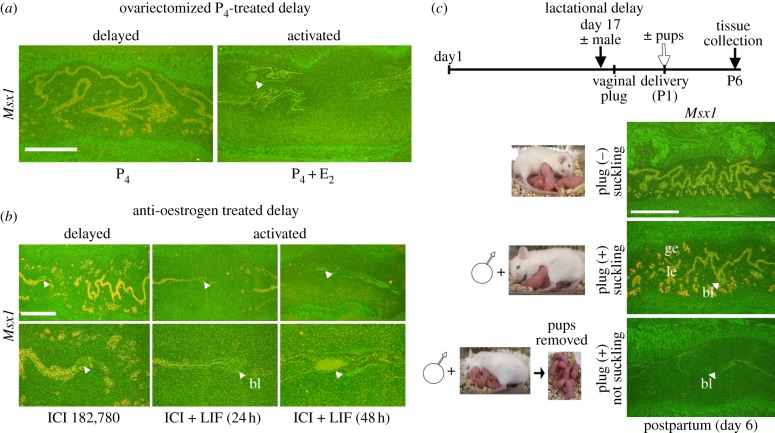
Persistent uterine expression of *Msx1* under different inducers of delayed implantation in mice. (*a*) Ovariectomized, delayed implanting mice treated with P_4_ or P_4_ + E_2_ were killed on day 8 of pregnancy. *In situ* hybridization showed distinct *Msx1* expression in luminal and glandular epithelia during delay with rapid downregulation following attachment reaction and embryonic activation 24 h after an E_2_ injection. (*b*) Oestrogen receptor antagonist (ICI 182,780) injected on days 3 and 4 induced delay, and LIF was injected twice on day 6 to terminate delay. *In situ* hybridization of uterine sections examined 24 or 48 h later showed persistent *Msx1* expression in delayed conditions which became undetectable with implantation initiation. (*c*) Females on day 17 of pregnancy were housed with males and checked for vaginal plug on days 19–20. Upon delivery (P1), pups were either removed (white arrow) or kept with the mother; uteri were collected six days postpartum (P6). Delayed implanting, lactating mothers had high uterine epithelial *Msx1* expression, which became undetectable following implantation upon withdrawal of suckling stimulus. Arrowheads denote embryos (bl). ge, glandular epithelium; le, luminal epithelium. Scale bar, 500 µm.

Ovariectomy of pregnant female mice prior to preimplantation oestrogen secretion on day 4 induces delayed implantation, and blastocyst dormancy can be maintained for several days to weeks with continued P_4_ treatment [22–24]. Blastocyst reactivation and implantation will not occur unless initiated with an injection of E_2_ or its downstream target LIF [5]. *Msx1* expression persisted in these delayed implanting females beyond its tightly regulated, transient expression on day 4 followed by rapid downregulation approaching attachment reaction upon activation by E_2_ in a P_4_-primed uterus (figure 1*a*).

We next addressed whether the expression of *Msx1* persists in delayed uteri induced by different approaches. Delayed implantation was induced in WT females by administration of a specific anti-oestrogen (ICI 182,780) on day 3 afternoon prior to preimplantation ovarian oestrogen secretion on day 4 morning to block oestrogen action; they were then killed on day 8. We found that under this condition *Msx1* expression persisted in the luminal and glandular epithelium (figure 1*b*). To determine whether LIF supplementation would initiate reactivation of dormant blastocysts and implantation, LIF (50 µg per mouse) was intraperitoneally injected at 1000 and 1600 h on day 7 in these anti-oestrogen-treated delayed implanting mice. LIF initiated implantation in these anti-oestrogen-treated delayed mice. Implantation sites were then examined for *Msx1* expression 24 and 48 h later by *in situ* hybridization. Strikingly, epithelial *Msx1* expression was progressively downregulated from the implantation site by 24 h to undetectable levels by 48 h (figure 1*b*).

Finally, we explored whether uterine *Msx1* expression persists in mice with lactational delay, a natural delay induced by suckling pups following postpartum mating; removal of the suckling stimulus promptly initiates embryonic activation and implantation. Pregnant females were housed with fertile males just prior to parturition. Mating was confirmed by the presence of a vaginal plug, and suckling was allowed to continue for 6 days in plug-positive females. We found that these lactating mice showed delayed implantation with unimplanted dormant blastocysts recovered from their uteri. Again, *Msx1* was distinctly expressed in the epithelium of these delayed uteri. More importantly, uterine *Msx1* expression became undetectable in those postpartum mated females from which pups were removed following delivery (figure 1*c*). Collectively, delayed implantation conferred by surgical (ovariectomy), pharmacological (anti-oestrogen) or natural (lactation) approaches consistently exhibited heightened uterine *Msx1* expression with rapid downregulation upon blastocyst activation and implantation.

### 4.2. Mice with uterine inactivation of *Msx* genes fail to achieve true delay and show compromised blastocyst survival

**Figure 2.**
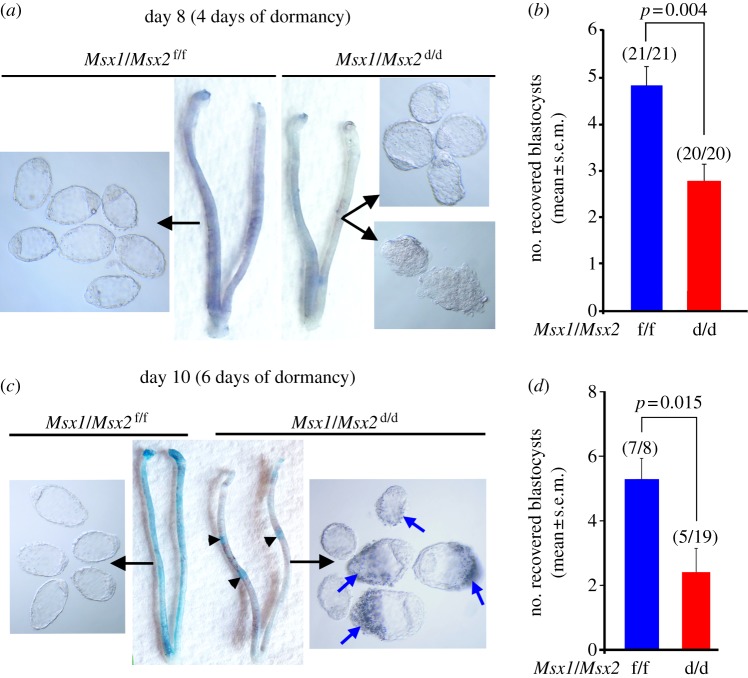
Uterine deletion of *Msx* genes fails to manifest true delay with reduced embryo survival. (*a*) Number of dormant blastocysts recovered from ovariectomized, P_4_-treated *Msx1/Msx2*^d/d^ females was significantly lower compared with floxed littermates (*Msx1/Msx2*^f/f^) on day 8 under delayed conditions. Several blastocysts recovered from deleted females showed signs of degeneration and poor morphological appearance compared with those recovered from floxed littermates. (*b*) A further reduction in number of recovered blastocysts was observed in deleted females when delay was extended to 10 days of pregnancy, and in some blastocysts, luminal epithelial cells were attached to the abembryonic trophectoderm (blue arrows). A subset of P_4_-treated, *Msx1/Msx2*^d/d^ uteri showed small swellings with faint blue bands at the site of blastocysts on day 10 (pseudo-implantation sites, arrowheads).

**Figure 3.**
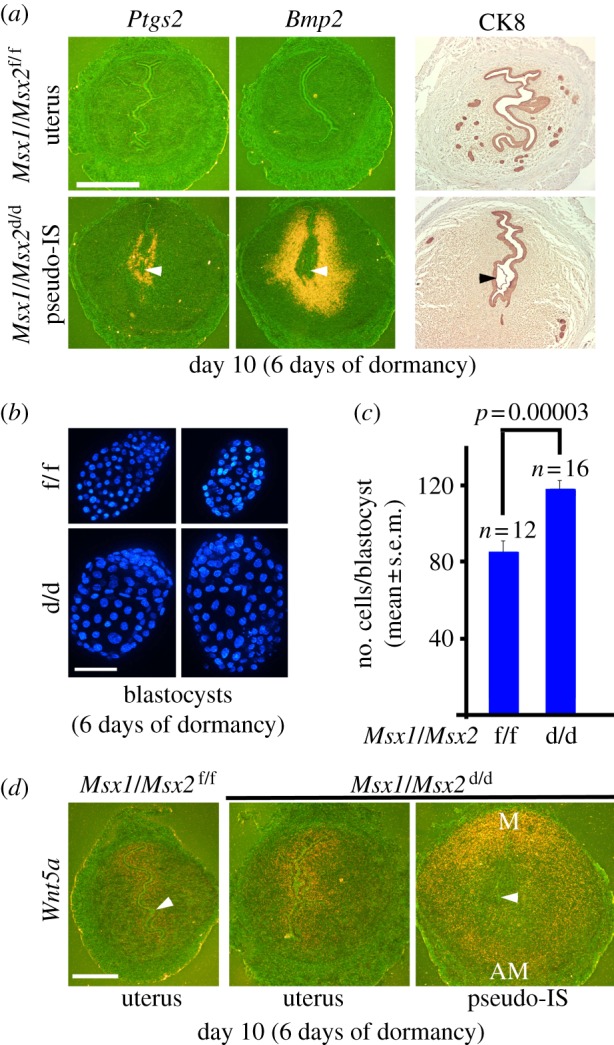
Blastocysts in *Msx1/Msx2*^d/d^ females under delayed conditions show implantation-like responses (pseudo-implantation) without epithelial invasion and display increased cell proliferation. (*a*) Pseudo-implantation sites (pseudo-IS) in *Msx1/Msx2*^d/d^ females on day 10 under dormant conditions show increased epithelial and stromal *Ptgs2* expression, and stromal *Bmp2* at the site of blastocyst without sign of trophectoderm invasion through the intact epithelial barrier as demarcated by cytokeratin 8 (CK8) staining. Arrowheads denote blastocysts. Scale bar, 250 µm. (*b*,*c*) Blastocysts recovered from *Msx1/Msx2*^d/d^ females appeared larger with higher cell numbers compared with those recovered from *Msx1/Msx2*^f/f^ delayed uteri. Blue, nuclear staining. Scale bar, 50 µm. (*d*) P_4_-treated delayed implanting *Msx1/Msx2*^d/d^ uteri and pseudo-implantation sites showed increased uterine *Wnt5a* expression, particularly at the site of blastocyst, compared with floxed littermates. Arrowheads denote blastocysts. Scale bar, 125 µm. M, mesometrial pole; AM, anti-mesometrial pole.

Delayed implantation occurs only if blastocyst dormancy is synchronized with uterine quiescence, and conversely, implantation can resume if blastocyst activation is superimposed on uterine receptivity. The persistent uterine *Msx1* expression during delay and its downregulation following blastocyst activation with initiation of implantation suggested that Msx1 is critical for synchronized quiescence of both the uterus and blastocyst. Although *Msx1* is primarily expressed in the uterus, *Msx2* is expressed as a compensatory partner in a similar manner as *Msx1* in the uterus lacking *Msx1* [7]. To obtain genetic evidence whether uterine *Msx1* and *Msx2* influence embryonic diapause under delayed conditions, we used mice with uterine inactivation of both *Msx1* and *Msx2* (*Msx1/Msx2*^d/d^) achieved by *Cre recombinase* expression driven by the progesterone receptor promoter. We found substantially fewer dormant blastocysts in ovariectomized, P_4_-primed *Msx1/Msx2*^d/d^ females on day 8 (4 days of dormancy) compared with littermate *Msx1/Msx2*^f/f^ (control) females under similar treatment conditions; many blastocysts recovered from *Msx1/Msx2*^d/d^ uteri showed signs of abnormal morphology and degeneration. Thus, uteri lacking *Msx* genes subject to dormancy conditions are not conducive to blastocyst survival (figure 2*a,b*). When the delay was extended to 6 days (day 10 of pregnancy), blastocyst recovery was further reduced. In addition, several females examined yielded no blastocysts (figure 2*c,d* and electronic supplementary material, table S1). The initiation of blastocyst attachment can be detected by an intravenous injection of a blue dye, because there is increased endometrial vascular permeability at the site of attachment [19,20]. Intriguingly, under ovariectomized, P_4_-treated delayed conditions, a small number of *Msx1/Msx2*^d/d^ females (33%) showed faint blue bands with small swellings. These swellings were not due to incomplete ovariectomy, as their size was reminiscent of day 5 or 6 of normal pregnancy, not day 10 (figure 2*c* and electronic supplementary material, figure S1*a,b*). Blastocysts were intimately apposed to the luminal epithelium with some recovered blastocysts having epithelial cells adhered to the trophectoderm (figure 2*c*, blue arrows). We also found some signs of decidualization (stromal cell proliferation) at the site of blastocyst with induction of *Ptgs2* (Cox2) expression, an early marker of vascular permeability and decidualization, similar to that seen on day 5 of normal pregnancy (figure 3*a* and electronic supplementary material, figure S1*c*) [21]. *Bmp2*, critical for decidualization [19,25], was also expressed at the same site, again reminiscent of expression at the implantation site on day 5 of normal pregnancy (figure 3*a* and electronic supplementary material, figure S1*c*). However, the luminal epithelium in *Msx*-deleted uteri was intact as shown by cytokeratin staining with no signs of trophectoderm invasion (pseudo-implantation; figure 3*a*). By contrast, delayed *Msx1/Msx2*^f/f^ females showed features of true dormancy with absence of blue bands, no decidual swellings and undetectable levels of *Bmp2* and *Ptgs2* at the site of blastocyst; an expected number of dormant blastocysts were recovered from uteri of these mice. As anticipated, *Msx1/Msx2*^d/d^ uteri fail to resume implantation by an E_2_ injection in contrast to floxed mice (see the electronic supplementary material, figure S2 and table S2). Collectively, the findings provide genetic evidence that Msx is critical for regulating the uterine milieu to confer uterine quiescence and blastocyst dormancy and viability.

It is known that dormant blastocysts exhibit little or no mitotic activity and cell proliferation [2,26]. Instead, we found that blastocyst growth was still active in *Msx1/Msx2*^d/d^ uteri with increasing blastocyst cell numbers when compared with blastocysts recovered from *Msx1/Msx2*^f/f^ uteri (figure 3*b*,*c*). These results provide evidence that blastocyst dormancy is under maternal control, because blastocysts failed to undergo complete dormancy owing to an altered uterine environment in *Msx1/Msx2*^d/d^ females.

To further explain the asynchronous activities between the blastocyst and uterus in the *Msx1/Msx2*^d/d^ females, we examined the status of *Wnt5a* expression in ovariectomized P_4_-treated, floxed and deleted females, because *Wnt5a* is a transcriptional target of Msx in the uterus during implantation and is upregulated in *Msx*-deleted uteri [7,27]. On day 10 under dormant conditions, *Msx1/Msx2*^d/d^ uteri showed higher levels of *Wnt5a* expression in the epithelium and subepithelial stroma when compared with *Msx1/Msx2*^f/f^ uteri under true delay, and *Wnt5a* expression was markedly upregulated in the pseudo-implantation sites at the mesometrial side in *Msx1/Msx2*^d/d^ uteri (figure 3*d*). *Wnt5a* expression at these sites reflected the induction of its expression in activated uteri undergoing implantation after LIF or E_2_ administration (see the electronic supplementary material, figure S3). These results suggest paracrine signalling by Wnt5a to the blastocyst. Taken together, these findings provide evidence that *Msx1/Msx2*^d/d^ uteri fail to achieve quiescence, leading to discordant blastocyst activation and ultimately demise of the embryo.

### 4.3. Oestrogen and P_4_ are not the primary inducers of *Msx1* in the mouse uterus

The above results raised questions whether *Msx* genes are regulated by P_4_ and/or oestrogen, because these hormones are prime endocrine factors involved in embryonic diapause and activation. To determine whether *Msx1* expression is regulated by ovarian hormones, ovariectomized *Msx1*^LacZ^ reporter mice were treated with vehicle (sesame oil), P_4_ (2 mg), E_2_ (25 ng) or P_4_ + E_2_ for 24 h. Surprisingly, *Msx1* was distinctly expressed in luminal and glandular epithelia in vehicle-treated uteri, and levels of expression were comparable among different treatment groups (see the electronic supplementary material, figure S4*a*). *Msx1* expression persisted in *Lif^−^*^/*−*^ uteri under similar treatment, whereas LIF injection in *Lif^−^*^/*−*^ females induced implantation with downregulation of uterine *Msx1* expression (see the electronic supplementary material, figure S4*b*). These results suggest that regulation of *Msx1* is not directly under the influence of E_2_ and/or P_4_ in mice.

We also found that exogenous P_4_ administration on days 3 and 4 of pregnancy did not rescue implantation failure in *Msx1/Msx2*^d/d^ females, although similar P_4_ treatment did not interfere with normal implantation in littermate floxed females (see the electronic supplementary material, figure S5*a,b*). This is consistent with comparable P_4_ responsiveness in floxed and deleted mice as assessed by expression of two P_4_-responsive genes *Indian hedgehog* (*Ihh*) and *Hoxa10* on day 4 of pregnancy (see the electronic supplementary material, figure S5*c*). By contrast, Ki67 and phospho-histone immunostaining results suggest that luminal epithelial proliferation persisted in deleted uteri on day 4, as opposed to little to no signal in the fully differentiated epithelium in day 4 floxed uteri (see the electronic supplementary material, figure S5*d*). These results suggest that incomplete luminal epithelial differentiation in the deleted uteri prevented receptivity towards blastocyst attachment.

### 4.4. *MSX1* is highly expressed during diapause in the North American mink

**Figure 4.**
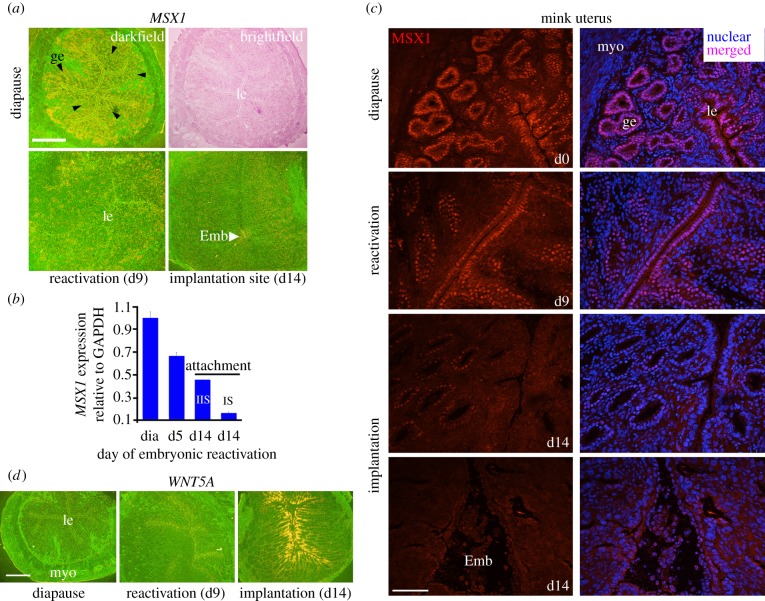
*MSX1* is highly expressed in diapausing mink uterus (*Neovison vison*) and downregulated upon embryonic reactivation with *WNT5A* expression in a reciprocal manner. (*a*) *In situ* hybridization results show distinct *MSX1* expressed in luminal and glandular epithelia in the diapausing uterus. Its downregulation was seen on day 9 of reactivation (d9) followed by undetectable levels at the implantation site on day 14 (d14). Arrowheads indicate glands. Scale bar, 500 µm. (*b*) qPCR results show downregulation of *MSX1* approaching attachment. IIS, interimplantation site; IS, implantation site. (*c*) Immunofluorescence shows MSX1 nuclear localization during diapause with reduced signals on day 9 of reactivation and undetectable signal at the implantation site. Scale bar, 100 µm. (*d*) *In situ* hybridization results show undetectable *WNT5A* expression in the diapausing uterus, and modest expression in luminal and glandular epithelia on day 9 of reactivation. By day 14, *WNT5A* is robustly expressed in the luminal epithelium and subepithelial stroma. Scale bar, 500 µm. d0, diapause; Emb, embryo; myo, myometrium; ge, glandular epithelium; le, luminal epithelium.

The results in mice described above prompted us to examine whether *MSX* genes play roles in other diapausing mammals, in particular, the American mink (*N. vison*) and Australian tammar wallaby (*M. eugenii*). The American mink is one of many mustelid carnivores that undergo seasonal obligate delay, which can persist for more than nine months in some species [1,4]. Although embryos develop into blastocysts, their further growth is arrested. Embryo reactivation can be triggered by prolactin administration with increased P_4_ output and uterine polyamine synthesis, with implantation occurring 12 days later [1,28]. We found increased *MSX1* expression in luminal and glandular epithelia of diapausing mink with reduced expression on day 9 after embryonic reactivation, followed by undetectable levels at implantation sites on day 14 (figure 4*a*). This expression pattern corroborates qPCR and immunofluorescence results (figure 4*b,c*). Intriguingly, uterine *WNT5A* expression in the mink showed an inverse relationship with *MSX1* expression similar to that seen in mice: *WNT5A* expression was undetectable in the diapausing uterus and correlated with higher uterine *MSX1* expression. By contrast, diminishing expression of *MSX1* was reflected in higher *WNT5A* expression. It was modestly expressed in the luminal epithelium and subepithelial stroma upon embryonic reactivation on day 9 but was robustly induced in these cell types upon implantation on day 14 with gradual tapering of expression towards the muscular layers (figure 4*d*). Hybridization with sense probes for *MSX1* or *WNT5A* did not show any positive signals (see the electronic supplementary material, figure S6*a,b*); *MSX2* was not detected by RT-PCR, although these primers could detect compensatory *Msx2* expression in mouse uteri deleted of *Msx1* (data not shown) [7].

### 4.5. *MSX2* expression persists during diapause in the Australian tammar wallaby

**Figure 5.**
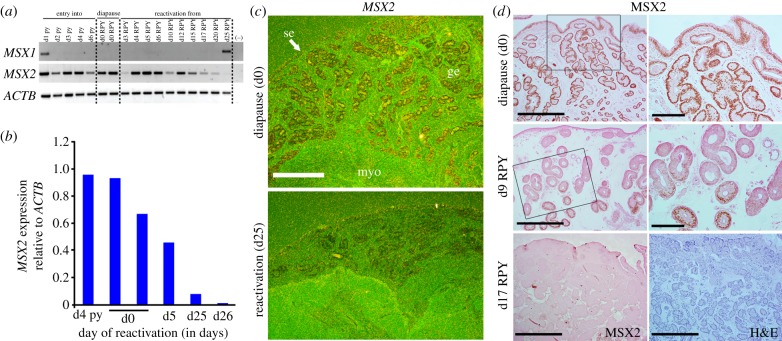
*MSX2* expression persists in diapausing tammar wallaby uteri (*Macropus eugenii*) and is progressively downregulated upon embryonic reactivation. (*a*) Representative RT-PCR results of *MSX1*, *MSX2* and *ACTB* at various stages of diapause and reactivation showed that *MSX2* expression remains high during diapause (d0, diapause; 68 and 145 days postpartum) with negligible expression of *MSX1*. Embryo reactivation by RPY showed gradual decreases in uterine *MSX2* expression approaching embryonic attachment (day 17 RPY) and towards delivery (days 25–26 RPY). py, pouch young indicating days postpartum. (*b*) qPCR results showed heightened *MSX2* expression relative to *ACTB* at diapause with gradual decreases during reactivation. (*c*) *In situ* hybridization results showed sustained *MSX2* expression in epithelia during diapause with downregulation on day 25 RPY. ge, glandular epithelium; se, surface epithelium; myo, myometrium. (*d*) Immunohistochemistry showed distinct nuclear localization of MSX2 in glandular and surface epithelia during diapause with gradual reduction on day 9 of reactivation from the surface epithelium and underlying glandular epithelium, but expression persisted in the deeper glands (left panels, scale bars, 250 µm; inset in right panels, scale bars, 100 µm). No positive signal was detected by day 17 of reactivation (bottom left panel). H&E staining on day 17 (bottom right panel). Scale bars, 250 µm.

The tammar wallaby undergoes a period of lactational delay during the first half of the year (February–June in the Southern Hemisphere) and a seasonal diapause after the winter solstice in June, thereby maintaining diapause for 11 months of the year [3,29,30]. In the tammar, the long duration of diapause, which can be extended further after the removal of the ovary [31], requires a supportive uterine environment because its secretory activity is minimal during diapause [13]. Remarkably, reactivation occurs synchronously among all pregnant wallabies during the summer solstice, the longest day of the year (22 December) with birth occurring 25–30 days later (approx. 22 January). Mating occurs within 1 h of birth, and the new conceptus reaches the 80-cell blastocyst stage to enter diapause [3]. Meanwhile, the newborn young remains in the mother's pouch for about nine months for the remainder of development and its nursing prevents reactivation of the blastocyst formed at the postpartum mating; RPY allows embryonic reactivation and attachment. We found that *MSX1* was undetectable in the pregnant uterus throughout diapause. By contrast, RT-PCR and qPCR results showed that *MSX2*, which has overlapping function with *MSX1*, was highly expressed during diapause with expression progressively diminishing after embryonic reactivation up to day 5 after RPY, reaching lowest levels after attachment to the endometrium which normally occurs on day 18 after RPY (figure 5*a,b*). Expression was undetectable approaching parturition on day 25 prior to birth on day 26.5. Again, both *MSX2* mRNA and MSX2 protein were highly expressed in the glandular and surface epithelia during diapause (figure 5*c,d*). Hybridization with a sense probe did not exhibit positive signal (see the electronic supplementary material, figure S6*c*). Intriguingly, MSX2 expression upon embryonic reactivation first becomes undetectable in the surface epithelium and superficial epithelial glands, with deeper glands still expressing MSX2 on day 9 of reactivation with levels becoming undetectable by day 17 (one day before blastocyst attachment; figure 5*d*).

## 5. Discussion

Although endocrine directives for embryonic diapause and reactivation have been described in several mammalian species [1,2], local molecular mechanisms that instruct the initiation and maintenance of diapause remain unknown. *Msx* genes are highly conserved across Metazoa from sponges to vertebrates and are considered ancient among homeobox gene families [8]. Our findings provide the first evidence linking Msx1 and Msx2 to the regulation of embryonic diapause. Of particular interest is the finding that the *Msx* expression pattern is conserved across distantly related taxa and between facultative (lactational) and obligate (seasonal) diapause. Although different species use distinct strategies to confer uterine quiescence synchronized with embryonic diapause to defer implantation, a recent interspecies embryo transfer study shows that embryos from non-diapausing sheep can enter diapause when placed in a delayed implanting mouse uterus and can be reactivated to produce normal offspring upon transfer back into the donor uterus [32]. This finding again implicates maternal regulation of embryonic diapause and suggests that embryos from non-diapausing species have the capacity to enter diapause if the maternal environment is conducive to this event. It further suggests that diapause is an ancient and conserved reproductive strategy and that all mammals may be capable to undergo diapause given the appropriate maternal regulatory signals [32]. Whether humans and their close primate relatives have or once had the potential to undergo delay remains uncertain [33]. Interestingly, gene array results show that *MSX1* and *MSX2* are expressed in the human endometrium in a similar manner to that in mice, with higher expression prior to and downregulation during the window of implantation (mid-secretory phase) [34,35]. These results may intrigue investigators to explore the status of uterine *Msx* expression in other diapausing and non-diapausing species.

Our studies in mice provide genetic and molecular evidence that *Msx* genes are critical to the initiation and maintenance of embryonic diapause, and uterine readiness to confer blastocyst reactivation and implantation. Increased *Wnt5a* expression in *Msx1/Msx2*^d/d^ uteri may function as a paracrine signal to promote proliferation of blastocysts entrapped within the uterine lumen without invasion, leading to their progressive demise within the hostile environment in *Msx*-deleted uteri. This is a potential explanation for the lack of true blastocyst dormancy in *Msx*-deficient uteri, because we have previously shown *Wnt5a* as a transcriptional target of uterine Msx [7]. Interestingly, we found increased *WNT5A* expression in the mink uterus after reactivation and implantation with diminishing *MSX1* expression similar to the pattern in mice, suggesting this inverse relationship between Msx1 and Wnt5a is conserved in the mouse and mink. It remains to be seen whether *WNT5A* in the tammar wallaby endometrium is also inversely expressed with *MSX2*.

Although P_4_ can prolong blastocyst survival under delayed conditions in mice, diapausing blastocysts of all three species can still survive for many days after ovariectomy without any ovarian hormone support [22,31,36,37]. Notably, our results show that *Msx1* expression persists even in uteri of ovariectomized mice without any steroid hormone treatment. It would be interesting to know whether blastocysts survival and implantation competency is coincident with *MSX* expression in ovariectomized pregnant mink and tammar wallaby uteri. Nonetheless, uteri in these three species require P_4_ priming and/or oestrogen for blastocyst reactivation (implantation competency) and implantation [38].

**Figure 6.**
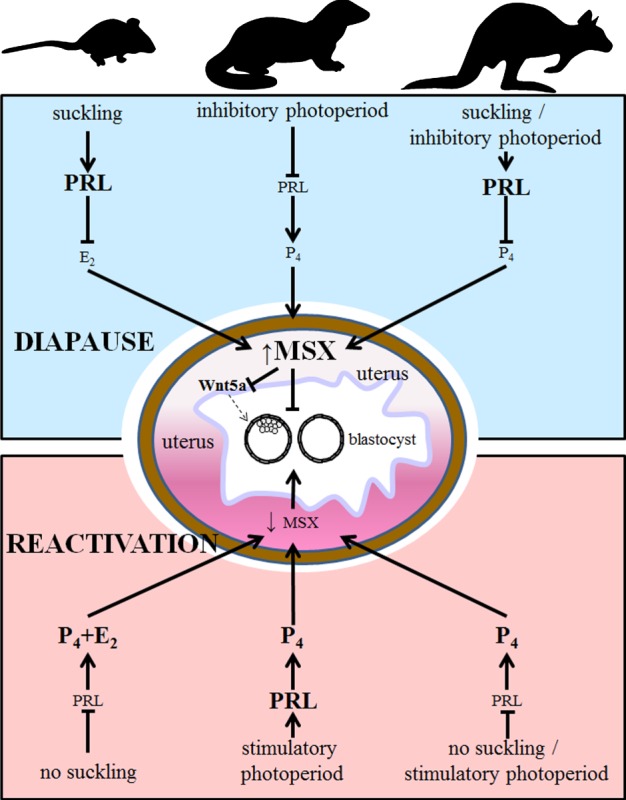
Potential schematic of the regulation of diapause in mouse, mink and wallaby. Although there is variation in the proximate stimuli used to induce diapause in these species in relation to differing roles of prolactin (PRL), progesterone (P_4_) and oestradiol-17β (E_2_), these diverse pathways ultimately converge to work through MSX in the uterus, which controls diapause and reactivation of the blastocyst, with an inner cell mass in eutherian mammals but not in marsupials. Genetic and molecular studies in mice show a lack of true diapause in the absence of uterine *Msx* genes potentially via upregulation of Wnt5a as a paracrine regulator for blastocyst growth, precluding fully fledged dormancy. Larger font size indicates higher levels of hormones.

Msx proteins are known transcriptional repressors in various developmental processes in association with the polycomb repressive complex [39,40]. Our findings of pseudo-implantation with induction of *Ptgs2* and *Bmp2* at the site of blastocyst without trophectoderm invasion through the luminal epithelium in *Msx*-deleted mouse uteri on day 10 under an experimentally induced delayed condition indicate a state of compromised transcriptional repression. This is consistent with uterine and blastocyst quiescence seen with persistent uterine expression of *Msx1* in mice and mink and *MSX2* in tammar wallaby during diapause. A deeper molecular understanding of synchronized uterine quiescence with blastocyst dormancy regarding potential upstream regulators and downstream targets that function through Msx in diapause in various species will require in-depth investigation. Nonetheless, our study has identified *Msx* genes as a common molecular link across three species representing different orders with diverse reproductive strategies (figure 6).

## 6. Acknowledgements

The authors thank Susanne Tranguch for some *in situ* hybridization experiments. We thank members of the tammar wallaby and mink research groups for help with the animal handling and field collections. This work was supported in part by NIH grants (nos. HD12304, HD068524 and DA06668 to S.K.D.), Australian Research Council grants (to G.S. and M.B.R.) and a NSERC Canada grant (no. 137013 to B.D.M). J.C. is supported by an NIH NRSA Fellowship (F30AG040858) and the University of Cincinnati MSTP (T32 GM063483). X.S. is supported by a Lalor Foundation Postdoctoral Fellowship.

## Supplementary Material

OpenBiology Dey Supplementary Data
