# Supplementary material for: A new role for muscle segment homeobox genes in mammalian embryonic diapause
Source: Open Biol. 2013 Apr;3(4):130035. doi: 10.1098/rsob.130035 (PMC3718335; doi:10.1098/rsob.130035)
Supplement: OpenBiology Dey Supplementary Data [file rsob130035-s1.docx]

**
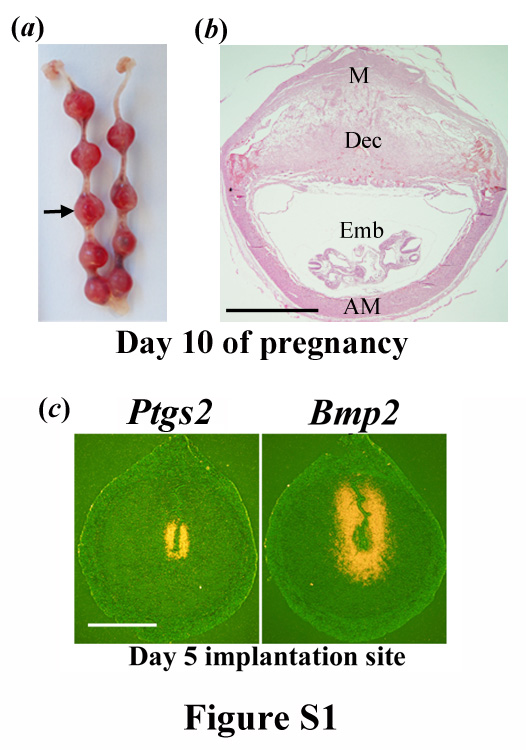
**

**Figure S1. Representative images of the uterine horns and section through an implantation site on day 10 of normal pregnancy, and *Ptgs2* and *Bmp2* expression in implantation sites on day 5 of normal pregnancy.** (**a**) Day 10 of pregnancy (arrow, implantation site). (**b**) H&E stained section. Dec, decidua; Emb, embryo; AM, anti-mesometrial pole; M, mesometrial pole. Bar, 1mm. (**c**) In situ hybridization of *Ptgs2* and *Bmp2* implantation sites recovered from day 5 of normal pregnancy in *Msx1/Msx2*^f/f^ mice. Bar, 500µm.

**
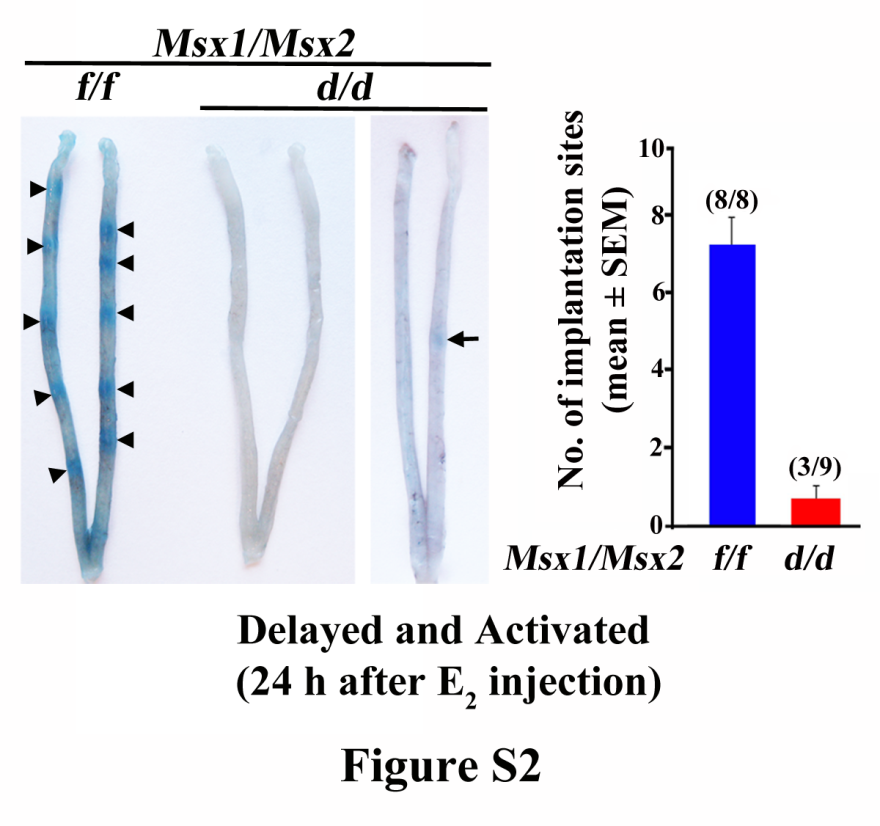
**

**Figure S2. E_2_ fails to initiate implantation in ovariectomized, P_4_-treated *Msx1/Msx2*^d/d^ mice under delayed conditions.** While 10 ng E_2_ initiated implantation in delayed *Msx1/Msx2*^f/f^ (blue bands, arrowheads), it failed to do so in deleted females. However, a small number of pseudo-implantation sites with weak blue bands were occasionally noted in *Msx1/Msx2*^d/d^ females under this condition (arrow, 3/9 females), and very few blastocysts were recovered from these females.


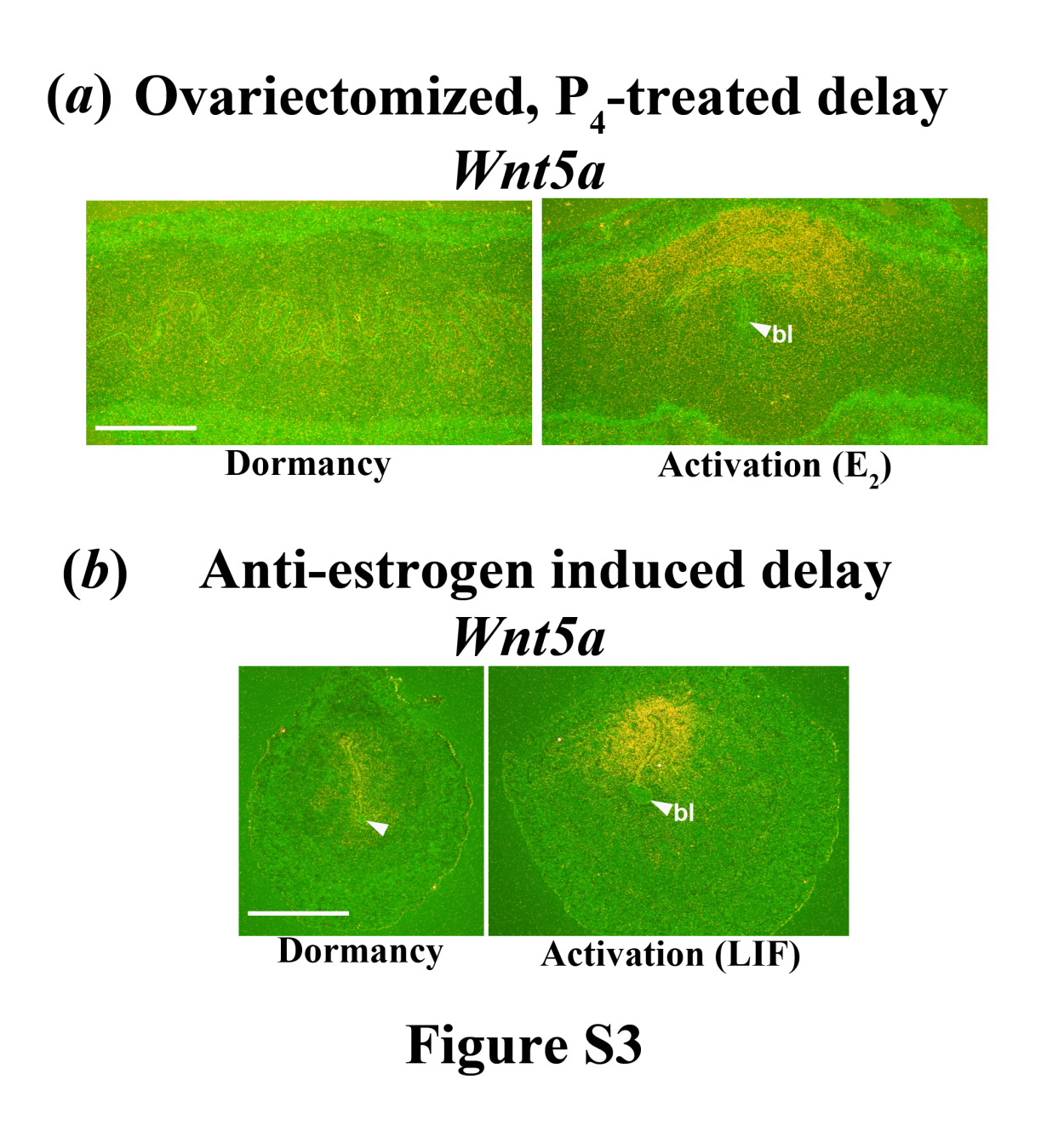


**Figure S3. *Wnt5a* is expressed at the mesometrial pole of the uterus upon exit from diapause by E_2_ or LIF activation.** (**a**) Ovariectomized, P_4_-treated delayed implanting WT females exhibit basal expression of *Wnt5a* is the luminal epithelium and subepithelial stroma of the uterus, which is rapidly induced at the mesometrial pole at the site of blastocyst upon activation after E_2_ administration. (**b**) WT females induced to undergo delay by anti-oestrogen administration also exhibit basal *Wnt5a* expression but is induced at the mesometrial pole after activation by LIF. Arrowheads denote location of blastocysts. bl, blastocyst. Bars, 500 µm.

**
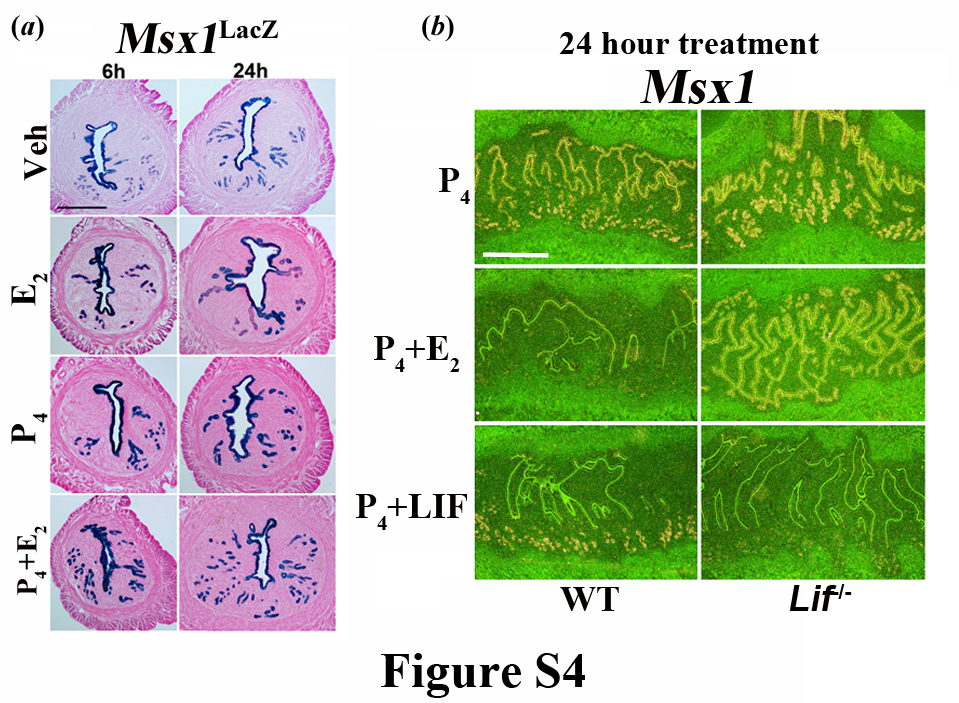
**

**Figure S4. P_4_ and/or E_2_ have minimal effects on uterine *Msx1* expression.** (**a**) *Msx1^LacZ^* reporter mice with knock-in LacZ were ovariectomized and subcutaneously administered 100 µL of sesame oil (control), 2 mg P_4_, 25 ng oestradiol-β (E_2_), or P_4_ + E_2_. Uteri were collected after 24 hours and processed for β-gal staining (blue), which showed similar expression pattern and intensity. (**b**) Ovariectomized, P_4_-treated WT or *Lif^-/-^* females showed sustained *Msx1* expression at 24 h. P_4_ + E_2_ treatment downregulated *Msx1* expression in WT females, but not in *Lif^-/-^* females; LIF administration downregulated this expression in *Lif^-/-^* females. Bars, 250µm.

**
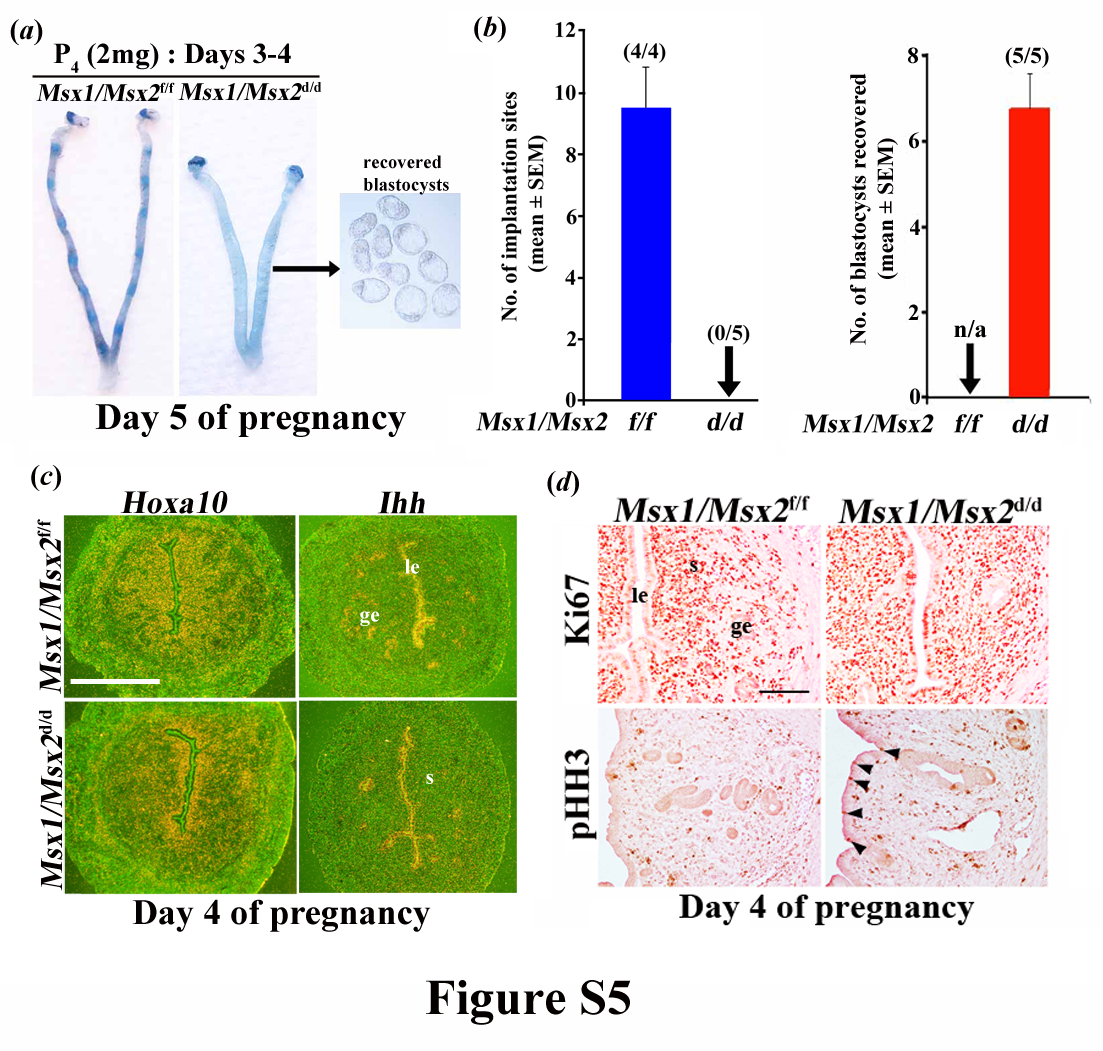
**

**Figure S5. Implantation failure seen in *Msx1/Msx2*^d/d^ females is not reversed by P_4_ treatment, and deletion of uterine *Msx* genes does not adversely impact expression of hormonally responsive genes critical for uterine receptivity, but does affect luminal epithelial cell proliferation.** (**a-b**), Pregnant *Msx1/Msx2*^f/f^ and *Msx1/Msx2*^d/d^ females were treated with 2 mg P_4_ on days 3 and 4 and examined on day 5 after blue dye injection. P_4_ administration did not rescue implantation failure seen in *Msx1/Msx2*^d/d^ females; unattached blastocysts were recovered from these females. (**c**) *Msx1/Msx2*^d/d^ females and control littermates were mated with WT males and sacrificed on day 4 of pregnancy. In situ hybridization shows comparable expression of P_4_-responsive genes *Hoxa10* and *Indian hedgehog* (*Ihh*) in the stroma and epithelium, respectively. Le, luminal epithelium; ge, glandular epithelium, s, stroma. Bar, 500µm. (**d**) *Msx1/Msx2*^f/f^ and *Msx1/Msx2*^d/d^ females were mated with WT males and uteri were collected on day 4 of pregnancy. Immunostaining for proliferation makers phospho-histone H3 (pHH3, G2/M phases) and Ki67 (G1, M, G2, S phases) were performed on formalin-fixed, paraffin embedded sections and counterstained with eosin. An increased number of cells with positive immunostaining was seen in *Msx1/Msx2*^d/d^ uterine epithelium. Arrowheads, positive pHH3 staining. Le, luminal epithelium, ge, glandular epithelium, s, stroma. Bar, 100µm.

**
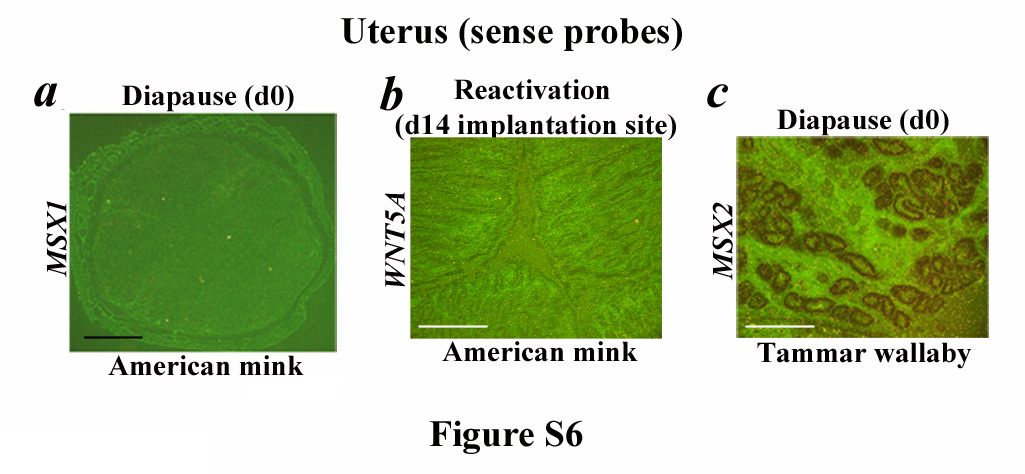
**

**Figure S6. In situ hybridization using sense probes in mink and wallaby uteri during diapause and reactivation.** Sense probes did not show any positive *MSX1* (**a**) or *WNT5A* (**b**) signals in mink uterus, or *MSX2* signals in tammar wallaby (**c**). Bars, 250 µm.

**Tables S1 to S2**

**Table S1. Blastocyst recovery and pseudo-implantation rates in P_4_-treated delayed implanting *Msx1/Msx2*^f/f^ and *Msx1/Msx2*^d/d^ females on day 10 of pregnancy.**

| **Genotype** | **No. of mice examined** | **No. females**  **without recovery of**  **blastocysts or pseudo-IS** | **No. of recovered blastocysts**  **(mean ± SEM)** | **No. pseudo-IS**  **recovered**  **(mean ± SEM)** |
| --- | --- | --- | --- | --- |
| ***Msx1/Msx2^f/f^*** | 8 | 1 | 5.3 ± 0.64 (7) | 0 |
| ***Msx1/Msx2^d/d^*** | 19 | 10* | 2.4 ± 0.75 (5) | 4.75 ± 0.85 (4) |

Littermate *Msx1Msx2*^f/f^ and *MsxMsx2*^d/d^ females were mated with WT males. Pregnant females were ovariectomized on day 4, treated with P_4_ (2 mg/mouse) from days 5-9. Pseudo-implantation sites (pseudo-IS) were recorded by the blue dye method on day 10. Uteri without pseudo-IS were flushed with saline to recover embryos. Numbers in parentheses indicate the number of females that gave blastocysts or pseudo-IS.

*blastocysts or pseudo-IS were recovered only in 9 of 19 *MsxMsx2*^d/d^ females examined on day 10 of pregnancy under delayed conditions; 10 females did not yield any blastocysts or pseudo-IS. For statistical analysis, we only used mice with recovered blastocysts and/or pseudo-IS (p<0.05, Student’s t-test).

**Table S2. Ovariectomized, P_4_-treated (delayed condition) *Msx*-deleted females show poor response to E_2_-induced implantation.**

| **Genotype** | **No. of mice examined** | **No. of mice with IS (%)** | **No. of blue bands**  **(mean ± SEM)** | **No. of blastocysts recovered** |
| --- | --- | --- | --- | --- |
| *Msx1/Msx2* ^f/f^ | 8 | 8 (100%) | 7.25 ± 0.73 | n/a |
| *Msx1/Msx2*^d/d^ | 9 | 3 (33%) | 0.67 ± 0.37^a^ | 0.78 ± 0.28 |

*Msx1Msx2^f/f^* and *MsxMsx2^d/d^* females were mated with WT males. Pregnant females were ovariectomized on day 4, treated with P4 (2 mg/mouse) from days 5-7. On day 7, they received an injection of E_2_ (10 ng/mouse). Implantation sites (IS) were recorded by the blue dye method 24 h later on day 8. Uteri without IS or faint blue bands were flushed with saline to recover embryos.

^a^Blue bands were very faint and marginally distinguishable from the remainder of the uterus, reminiscent of the pseudo-implantation sites found in dormant *Msx1/Msx2*^d/d^ females without oestrogen activation. Although blue bands were clearly evident in E_2_-activated *Msx1/Msx2* ^f/f^ females, the occasional faint blue bands seen in *Msx1/Msx2*^d/d^ females were included for analysis.

n/a, not applicable.
